# Chlorin, Phthalocyanine, and Porphyrin Types Derivatives in Phototreatment of Cutaneous Manifestations: A Review

**DOI:** 10.3390/ijms20163861

**Published:** 2019-08-08

**Authors:** Sarah Raquel De Annunzio, Natalia Caroline Silva Costa, Rafaela Dalbello Mezzina, Márcia A. S. Graminha, Carla Raquel Fontana

**Affiliations:** School of Pharmaceutical Sciences, São Paulo State University (Unesp), Araraquara, Rod. Araraquara-Jaú, km 01, Campus Ville, Araraquara 14800-903, São Paulo, Brazil

**Keywords:** photodynamic therapy, chlorins, phthalocyanines, porphyrins, skin diseases, skin rejuvenation

## Abstract

Recent scientific research has shown the use of chlorin, phthalocyanines, and porphyrins derivatives as photosensitizers in photodynamic therapy in the treatment of various pathologies, including some of the major skin diseases. Thus, the main goal of this critical review is to catalog the papers that used these photosensitizers in the treatment of acne vulgaris, psoriasis, papillomavirus infections, cutaneous leishmaniasis, and skin rejuvenation, and to explore the photodynamic therapy mechanisms against these conditions alongside their clinical benefits.

## 1. Introduction

Although the benefits of sun exposure in promoting health and the application of light as a therapy for the treatment of cutaneous disorders in combination (or not) with photosensitive molecules are well-known since the early civilizations of China, Greece, and Egypt [1], the first scientific research using photodynamic therapy (PDT) was performed only in 1900 by Oscar Raab, when he observed the photosensitive activity of acridine orange dye in *Paramecium caudatum*, when subjected to irradiation in the presence of oxygen, leading *Paramecium caudatum* to death [2]. In 1903 Hermann Von Tappeiner observed that the combination of eosin and light caused a reduction in skin diseases, such as condylomata, psoriasis, skin cancer, lupus vulgaris, and syphilis. It is now well established in the literature that the action of PDT depends on the absorption of visible light by a suitable photosensitive agent that will produce reactive oxygen species (ROS), which can destroy microorganisms, blood vessels, and cancer cells [3]; its application in the treatment of skin diseases has gained prominence and interest around the world. Although most skin disorders alone do not pose any threat to the life of patients, the growing demand for improvements in skin appearance, mostly associated with the patient’s self-esteem and quality of life is undeniable. Therefore, the continuous introduction of new light sources and photosensitizers (PS) associated with the easy access of the skin to photonic therapies contribute to the growing use of PDT for the treatment of various cutaneous conditions [4].

Topical application of PDT is an innovative therapeutic option and corresponds to the evolution of inflammatory disease treatment including acne vulgaris and psoriasis, or infectious diseases such as cutaneous leishmaniasis and verruca vulgaris. Moreover, the application of this therapy in skin rejuvenation has been widely studied [5,6].

The compounds chlorin, porphyrin, and phthalocyanine have been commonly used as PS, mainly due to their photophysical characteristics suitable for PDT application such as high quantum yield of singlet oxygen and the light absorption at 600–750 nm, contributing to the applications that require greater light penetration [7,8]. Additionally, there are some PS belonging to this group of molecules that are already in the market and are approved for clinical use [9,10,11] (Table 1).

## 2. Photodynamic Therapy and Photosensitizers

Briefly, a low-intensity visible light source, a non-toxic dye in the absence of light, i.e., a PS, and the presence of oxygen in the target cell or tissue are necessary for PDT. PS is excited by light in a specific wavelength, giving rise to two reactions (type I and the type II) that can occur simultaneously. In type I reactions, the excited triplet state can react with the biomolecules by transferring charges, resulting in radicals and radical ions that react with molecular oxygen, causing the production of ROS. In type II reaction, the direct energy transfer occurs from the PS in the triplet excited state to the oxygen in the fundamental triplet state, forming singlet oxygen, a highly cytotoxic form [12,13,14].

Recently, Scherer et al., 2017 [15] described the types III and IV mechanisms of photodynamic reactions that are not dependent on the presence of oxygen, allowing the occurrence of cytotoxic effects in intracellular structures. Type III photosensitive molecules are usually antioxidant carrier sensitizers (ACS), presenting properties that increase the generation of singlet oxygen and decrease the concentration of native free radicals in the target cells. For type IV, the authors suggest that their cytotoxic mechanism is related to the PS that are not able to bind to a molecular target, but under light excitation, an intramolecular remodeling occurs through a photoisomeration process, contributing to the binding of photosensitive molecule to its molecular target.

In order to be used in PDT, the PS must meet some criteria, such as low (or no) toxicity in the absence of light, high selectivity, non-prolonged photosensitivity, chemical purity, and low aggregation in solutions [16,17]. 

The following sections present the literature reports showing the use of PSs of the type chlorins, phthalocyanines, and porphyrins in the treatment of acne vulgaris, psoriasis, cutaneous leishmaniasis, and skin rejuvenation.

## 3. Acne Vulgaris

Acne vulgaris is a chronic inflammatory skin disease characterized by the excessive growth of the microorganism *Cutibacterium acnes*, formerly known as *Propionibacterium acnes,* in the sebaceous glands [18].

According to the Global Burden of Disease, acne affects approximately 50 million people in the United States annually and 600 million people around the world, ranking this skin disease as the eighth most prevalent disease in the world in 2012 [19,20,21]. Although acne is not a life threatening disease, different studies have reported the severe psychological effects in patients, which can affect sociability, cause phobias, and lead to depression symptoms [22,23,24].

Conventional acne treatments consist of the topical application of retinoids/benzoyl peroxide or antibiotics such as tetracyclines and isotretinoin, which can also be systemically administered [25,26,27]. However, these therapies can cause skin irritation and lead to poor patient compliance, as they require daily use [28]. Another important aspect refers to the prolonged use of antibiotics, which can be ineffective [29], contribute to the development of resistant microorganisms, and present several side effects requiring rigorous monitoring [30]. Therefore, the search for new, safe, and efficient therapeutic measures is necessary. In this context, PDT has been suggested as an alternative for treatment of acne. Several studies have shown that PDT is safe and effective for inflammatory and non-inflammatory acne lesions and can significantly improve these lesions [5,31]. Moreover, because ROS produced by PDT does not present any specific molecular target, it is easy to bypass microbial drug resistance, conferring advantage to this therapy over antibiotic treatments [32]. The efficiency of PDT against acne vulgaris are mainly due to the reduction of sebum produced by the sebaceous glands, reduction of *C. acnes* load, occlusion of the pilosebaceous orifice causing keratinocytes detachment, and reduction of scar formation [33,34,35].

In the past years, several in vitro and clinical studies have been developed mainly using 5-aminolevulinic acid (ALA) and methyl aminolevulinate (MAL) as PS in PDT in the treatment of acne [36,37]. However, recently, in vitro and in vivo studies using chlorins have also presented satisfactory results [38]. On the other hand, phthalocyanines, to our knowledge, have not been investigated to date for the treatment of this dermatosis. Table 2 shows the detailed studies using chlorins and porphyrins as PSs against acne vulgaris [37,38,39,40,41,42,43,44,45,46,47,48,49,50,51,52,53,54,55,56,57,58].

## 4. Psoriasis

Psoriasis is a skin condition that affects approximately 2.5% of the Caucasians, 1.3% of the African–American population, and 0.6% to 4.8% of the population worldwide [59]. Psoriasis has been traditionally considered as a common chronic inflammatory skin disease, which presents a broad spectrum of clinical phenotypes, triggered by an interplay of numerous factors such as environmental, immunological, and possibly genetic features, which can also be considered as participants in the development of this multifactorial disease [60]. The pathogenesis of the disease includes mainly the activation and migration of T cells to the dermis, triggering the release of TNF-α, IL-6, IL-1β, and IL-17, among other citokines, leading to an inflammatory response and increased proliferation of skin cells [61,62,63,64]. The disease treatment mainly relies on the therapeutic agents that modulate the immune system or normalize the keratinocyte differentiation program. The current available treatment are agents for topical use such as emollients, dithranol, tar, deltanoides, corticosteroids, tacrolimus, and ultraviolet radiation (UV) or for systemic use, including methotrexate, cyclosporine, acitrecine, hydroxyurea, fumarates, etc. [59]. Although its clinical efficacy, the use of combined therapy using psoralen and UVA (PUVA) [65] showed severe side effects such as nausea, headache, erythema, burns, bringing pain, and discomfort to the patient during the treatment, and consequently causing patient low compliance; besides, there is an increased risk of skin cancer development due to UVA irradiation [66]. Thus, the development of more specific, less toxic, and more bearable new therapeutic approaches is mandatory. 

It was reported that the use of ALA [67,68,69,70], a substrate for the biosynthesis of the photoactive protoporphyrin IX (PPIX), on PDT for the treatment of psoriasis causes apoptosis of the T-cells [67] and a decrease in the production of the main citokines related to the disease development [67]. Another study showed that patients undergoing ALA treatment followed by PDT had diminished lesions and inflammatory response [66]. The reduction in cell proliferation can be related to the activation of the MAPK pathway, promoting an increased level of expression of the apoptosis related genes *p38, JNK and ERK, PARP* and *caspase 3* [66]. Other studies have been conducted in order to identify new synthetic PS porphyrins to be applied on the treatment of this skin disorder [71,72]. 

The metallophthalocyanine ZnPc-F7, a phthalocyanine agent α-(8-quinolinoxy) zinc, is a metal coordinated compound presenting good solubility, low toxicity, and a strong effect on disease control. This metal-based compound is excited at 670 nm, allowing it to reach deeper layers of skin, besides its safeness when compared to PUVA-based treatment. The antipsoriasis effect of ZnPc-F7 reduced both HaCaT cell proliferation after light irradiation at 670 nm as well as the levels of IL-17, diminishing the psoriatic lesions [59,66]. 

Another PS that was active against skin inflammation is the silicon phthalocyanine (Pc) 4 coupled with red light. It has been shown that (Pc) 4 cause cell death when evaluated against cell lines derived from lymphoids (Jurkat) or epithelium (A431), highlighting singlet oxygen and ROS generation as possible inducers of cell death, acting in a dose-response way, i.e., the higher the concentration of the compound the greater the number of apoptotic cells [73]. The studies of phthalocyanines and porphyrins on PDT applied in the treatment of skin disorder are available in Table 3 [66,71,73,74,75].

## 5. Viral Warts Caused by Human Papillomavirus (HPV)

Warts are benign cutaneous lesions usually caused by human papillomavirus (HPV) infection [76]. According to the type and site, these viruses present varied clinical manifestations. Multiple flat warts occur on the face, which appear as flattened papules, commonly related to the HPV type 1 and 3. These warts negatively influence the aesthetics and sociability of patients [77].

The conventional modalities applied for the treatment of these warts are cryotherapy, electrosurgery, topical keratolytic agents, and antimiotics. Because the application is simple and safe and present rapid response, cryotherapy has been one of the most commonly used treatment modalities. However, relapse of warts after several sections of cryotherapy is very common and, alongside this treatment, hyperpigmentation and scars formation onto the local application is frequent [78]. Thus, the search for alternative modalities is extremely important.

Clinical studies have reported that ALA has shown positive results in the treatment of recalcitrant facial warts [79,80]. The literature suggests that ALA is absorbed and converted to PPIX by HPV-infected keratinocytes. The early stages of viral infection can be inhibited via PPIX-mediated PDT associated with 630 nm laser irradiation. This combination causes the host cells to form ROS by selectively inactivating non-enveloped viral particles [80]. 

Additionally, a study conducted by Morton et al. [81] suggests that the mechanism of action of ALA-PDT in warts is related to the selective photothermolysis of oxyhemoglobin in the microvasculature, causing damage in the dilated superficial capillaries present in these warts.

Table 4, depicts the studies that have been using porphyrin derivatives for the treatment of these warts [76,82,83,84,85,86].

## 6. Cutaneous Leishmaniasis

Leishmaniasis is a global problem, focusing on 98 developing countries especially those in Southeast Asia, East Africa, Latin America, and the Mediterranean with 12–15 million people infected and 350 million at risk of acquiring the disease [87], and is fatal if not treated properly.

The etiological agents of these diseases are trypanosomatids parasites belonging to the genus *Leishmania*, which are transmitted by the bite of sandflies. During the insect blood meal, promastigote forms of the parasite are inoculated into the mammalian skin [88]. The parasites are then engulfed mainly by macrophages, followed by differentiation to intracellular amastigotes, which multiply until the rupture of the infected cells [89,90].

Cutaneous leishmaniasis (CL) is the most common clinical manifestation of the disease characterized by the formation of papules that gradually progress to ulcers with an indurated border. Although most cutaneous leishmaniasis ulcers spontaneously heal over a long period that lasts months, atrophic and disfiguring scars create a substantial psychological burden for years to come. Depending on the patient immune response and the parasite species, the disease can progress to diffuse cutaneous leishmaniasis and/or mucosal leishmaniasis, leaving life-long scars and serious disability and stigma [91]. An estimated 0.7 to 1.2 million cases are reported per year worldwide, especially affecting Latin America and Middle East, with a total of 40 million people living with scars [92].

The current treatment for leishmaniasis are based on few chemotherapeutic options, which present several drawbacks, such as parenteral administration, severe side effects, and low efficacy [87,93]. The pentavalent antimonials, methylglucamine antimoniate (Glucantime^®^), and sodium stibogluconate (Pentostan^®^) are the first choice drugs for its treatment [88,94]. Other drugs, such as amphotericin B, pentamidine, and miltefosine, are the second choice drugs, but they also produce side effects that can endanger the patient’s life [95].

Miltefosine is the first oral drug that can be used in the treatment of leishmaniasis [96] and is reported as more effective than antimony for the treatment of CL in Bahia, Brazil [97]; however, treatment failure has been reported [98]. Thus, newer and safer therapeutical approach is mandatory. In this scenario, PDT has been evaluated. 

ALA has been investigated for the treatment of CL, commonly associated with red and blue light. ROS formed during photodynamic action causes parasite death necrosis or apoptosis-like mechanisms [99]. Data from the literature showed that porphyrins have affinity for cell membranes and in order to improve this property and increase the stability of the compounds, liposomes containing porphyrins have been used, thus promoting the internalization of the photosensitive agents and, consequently improving the efficiency of the photodynamic therapy [100].

Chlorin-e6 is another PS that has been studied and an in vitro study by Pinto et al., [101] described that PDT mediated by this PS affected *Leishmania major* viability; although these authors claimed that this PS is interfering in the parasite mitochondrial activity, other assays must be done in order to corroborate this hypothesis, such as O_2_ consumption, ATP production, and mitochondrial membrane potential evaluation, for instance. The authors further demonstrated that this PS accumulates in the cytosol, causing parasite morphological modifications. Although the authors claimed that the potential of this PDT therapy for the treatment of CL, the lack of assays involving the amastigotes cells, the forms responsible for the development of the disease, compromise this conclusion.

Another interesting approach that has been studied is the association of PDT and conventional antileishmanial drugs, and this type of strategy is also being applied for CL [102]. For instance, the phthalocyanine chlorosaluminum liposome compound (AlClPC) was developed through nanotechnology techniques for controlled drug release, improving the drug bioavailability and diminishing the drug toxicity; thus this PS was evaluated in association with miltefosine in an in vivo CL model. Although this promising therapeutic approach is able to decrease the parasite load in the site of infection and could be done without hospitalization, the authors did not investigate its toxicity to date [103].

Moreover, in order to control the disease, the principle of PDT has been explored in order to promote parasite photo-inactivation in vitro, especially in combination with porphyrins and phthalocyanines, and used them as vaccines. Indeed, Vianna and cols [104] demonstrated that photo-inactivated parasites used as vaccine may modulate the immune response of susceptible BALB/c mice, and prophylactics protecting them against challenging infections [104]. Table 5 shows the details of the parameters and results used in the studies related to PDT and cutaneous leishmaniasis [102,103,104,105,106,107,108,109].

## 7. Skin Rejuvenation

In addition to the application against inflammatory and infectious diseases, clinical studies have shown that the application of PDT has shown improvement in signs of skin aging [5].

Aging is characterized by the appearance of wrinkles, fine lines and small capillaries, as well as increased roughness and changes in cutaneous pigmentation [110].

In PDT, ALA, MAL, and chlorin-e6 have been used in the search for cutaneous rejuvenation. Studies suggest that the mechanism of PDT action for this purpose is associated with increased collagen type 1 and skin thickness [38,111,112]. Table 6 shows the studies that investigated the action of PDT on skin rejuvenation [111,112,113,114,115,116,117,118,119].

## 8. Discussion and Future Perspectives

The use of PDT allied to topical and nontoxic PSs has been explored in a number of different clinical applications not only for the treatment of inflammatory or infectious diseases, but also for skin rejuvenation [4]. This paper highlighted the clinical evidences and the usefulness of the three classes of PSs, chlorins, phthalocyanines, and porphyrins and their use in PDT and in fighting acne vulgaris, psoriasis, HPV-associated warts, and leishmaniasis. Moreover, PDT can open an avenue in fighting bacterial drug resistance as can be depicted in studies of PDT on helping in prolonged acne remission [4,30]. PSs used in studies to suppress acne are mostly derived from porphyrin, such as ALA and MAL [36,37], and more recently the number of studies using chlorin derivatives, for example chlorin-e6 [38], has been increased. To date, work using phthalocyanines for this dermatological condition has not been observed, possibly due to the fact that this condition does not require a greater tissue penetration of the light made possible by this class of PS.

The introduction of ALA as PS has been considered as a major advance of PDT in dermatology. ALA is metabolized to PPIX via the heme biosynthetic pathway, which is common to all nucleated mammalian cells [120]. The accumulated excess of PPIX generates ROS after illumination, leading to apoptosis and necrosis of target tissue. This precursor is small enough to penetrate the skin barrier and can accumulate inside pilosebaceous units and hyperproliferative keratinocytes compared to normal skin, making the treatment selective [121]. In addition, in many pathological skin lesions, because the stratum corneum barrier is generally compromised, this might be further explored in order to increase the selectivity action of ALA due to its facilitated penetration. An additional advantage of PPIX refers to its photodegradation during irradiation, which means that its selective cytotoxic effect continues only during PDT application. However, despite the efficiency of this precursor in acne treatment, studies have shown that this option is painful and cause erythemas, edemas, bubbles, and transient hyperpigmentation, which requires further studies in order to determine the right dosage of both light and PSs [122].

The prodrug MAL, an ALA derivative, is more lipophilic, allowing a greater tissue penetration than ALA. Although the MAL PS chlorin-e6 has shown interesting results in in vitro and in vivo models of acne vulgaris [4,38], further studies are necessary in order to confirm its efficacy, including studies on biofilms, which are poorly explored in the literature and will define better parameters for its implementation in clinical protocols.

As for psoriasis, studies were performed using porphyrin derivative such as ALA and phthalocyanine derivatives [66,67]. Studies using chlorin for this disorder have not been observed so far. The efficacy of PDT in the treatment of psoriasis is still controversial in the literature. Studies have shown that the use of ALA may be effective; however, it may show adverse effects making it difficult to be tolerated by patients [70]. Thus, studies involving lower concentrations of the drugs as well as the association with low doses of light and even the use of other PSs may be necessary for the success of PDT to be confirmed in the treatment of psoriasis.

In the treatments for warts caused by HPV infection, ALA also excels in photodynamic treatment due to the antiviral properties of this therapy [78]. Although PDT was successful in this application, pain during irradiation still hampers the widespread use of this treatment modality [123].

The main studies involving the use of porphyrins in the treatment of cutaneous leishmaniasis use ALA as PS, but a study conducted by Sah et al. [124] has reported that the ALA-mediated PDT antiparasitic mechanism is probably due to the death of the infected host cells, and not through the death of the parasite because of the inability of these pathogens to produce PPIX, since the literature has reported that *L. amazonensis* is deficient of most of the enzymes that are part of the biosynthetic pathway of the heme group. Thus, the use of PSs having direct action onto parasite is interesting. Indeed, PSs such as chlorins and phthalocyanine have been explored in PDT for cutaneous leishmaniasis treatment. Chlorin-e6 might impair the parasite mitochondrial function, leading to the parasite death, since this unique organelle is involved in many vital mechanisms, such as calcium homeostasis [125]. Another interesting approach is the combination of the PDT and the use of current antileishmanial drugs. In this context, Ribeiro et al. [103] developed an AlClPC and combined it with the drug miltefosine to evaluate the synergistic effect, which showed high leishmanicidal activity.

As for skin rejuvenation, the use of PDT in the area of esthetics has shown good results, showing an improvement in the appearance of the skin in the treated area and low side effects, causing much interest for the scientific community in clinical studies. Although the most employed PSs for this purpose used so far are the porphyrin derivatives ALA and MAL. A recent study [53] has shown that chlorin-e6-mediated PDT may be promising in cosmetic area since this therapy has been able to increase collagen I production, which may help both in the skin rejuvenation process as well as to minimize the scars caused by severe acne. Thus, chlorin-e6 can be in a near future a multi-task PSs, useful not only in facial rejuvenation, but also in the treatment of inflammatory diseases [38].

In summary, this review showed that although many reports have been published in recent years using PDT and its application in several skin-related diseases, new studies are need in order to understand the immune system response of the patient after the treatment, using more accurate assessment and standardization of pre-irradiation time, light dosimetry, and type of light source. Besides, PSs must have lower toxicity, being possible to evaluate studies with lower concentrations and larger numbers of photodynamic applications. Finally, the combination of PDT and nanotechnology will certainly improve the properties of these PSs as well as their action in photodynamic treatment, allowing the establishment of newer, safer, and more efficient protocols to be used in the clinical practice.

## Figures and Tables

**Table 1:** Photosensitizers approved for clinical use. In this review, we summarize the investigations of the last 15 years using chlorin, phthalocyanine, and porphyrin derivatives as PS in the treatment of acne vulgaris, psoriasis, papilloma virus infections, cutaneous leishmaniasis, and skin rejuvenation, and discuss the mechanisms of PDT against these conditions and observe their clinical actions.

PS Type	Product	Application
**Chlorin**	Foscan ^®^	Approved in Europe for head and neck cancer
	Laserphyrin ^®^	Approved in Japan lung cancer
**Porphyrin**	Photofrin ^®^	Approved in the United States for esophageal cancer; Approved in Japan for gastric cancer; Approved in Canada for recurrent bladder cancer
	Metvix ^®^	Approved in the United States and Europe for actinic keratosis
	Levulan ^®^	Approved in the United States and Europe for actinic keratosis
	Photogem ^®^	Approved in Russia for basal cell carcinoma
	Visudyne ^®^	Approved in China and the United States for macular degeneration
**Phthalocyanine**	Photosens ^®^	Approved in Russia and India for sarcoma and choroidal, eye, eyelid, cervical, bladder tumors

**Table 2:** Descriptions of the studies that used derivatives of porphyrins and chlorins for the treatment of acne vulgaris.

First Author and Year	Type of Study	PS	Chemical Family	Concentration (PS)	Pre-Irradiation Time	Light Source	Dose of Light	Results
Pollock et al., 2004	Clinical	ALA	Porphyrin	20%	3 h	Laser 635 nm	15 J/cm^2^	A statistically significant reduction in the count of inflammatory acne lesions was obviated after second application of ALA-PDT.
Hörfelt et al., 2006	Clinical	MAL	Porphyrin	160 mg/g	3 h	Red light 635 nm	37 J/cm^2^	Although MAL-PDT was effective in treating moderate to severe opposing facial acne, further studies are still needed.
Hörfelt et al., 2007	Clinical	ALA	Porphyrin	20%	3 h	Red light 635 nm	30, 50, 70 J/cm^2^	Of 15 patients, 8 presented clinical improvement after PDT. No significant reduction of *P. acnes* or sebum excretion after PDT was observed. Thus, the authors suggest that other mechanisms of action of PDT in acne may be involved that are not correlated to the eradication of *P. acnes* and decrease in sebum secreted by the sebaceous glands.
Hörfelt et al., 2009	Clinical	MAL	Porphyrin	160 mg/g	3 h	Red light 635 nm	15 J/cm^2^	Although PDT and phototherapy significantly decreased acne score, no significant difference was observed between the PDT group and the group that solely applied light. Fluorescence maps showed low selectivity for MAL-induced fluorescence in acneic lesions suggesting a mechanism of total and non-selective photoablation. No significant reduction of *P. acnes* and sebum excretion was found.
Bissonnette et al., 2010	Clinical	MAL	Porphyrin	80 mg/g	90 min	Red light	25 or 27 J/cm^2^	MAL-PDT without occlusion reduced the number of inflammatory lesions in patients with facial acne vulgaris.
Mal et al., 2013	Clinical	ALA	Porphyrin	5%	1 h	LED 633 nm	96–120 J/cm^2^	The parameters of PDT used in this study were ideal for treating different degrees of acne in Chinese patients, presenting higher activity for grade IV cystic acne and mild side effects.
Mei, Shi, and Piao 2013	Clinical	ALA	Porphyrin	10%	1 h	Intense pulsed light 420–950 nm	10–13 J/cm^2^	ALA-IPL-PDT was effective in the treatment of moderate to severe acne vulgaris, presenting mild to transient side effects.
Pinto et al., 2013	Clinical	MAL	Porphyrin	160 mg/g	90 min	Red light 635 nm	37 J/cm^2^	PDT presented faster action and better response than the red light alone and could reduce sebaceous gland size and cause acne remission in the long run.
Jeon et al., 2015	In vitro	Chlorin- e6	Chlorin	100 μg/ml	Uninformed	Halogen light	Uninformed	PDT was effective against inflammation caused by *P. acnes.*
Pariser, 2016	Clinical	MAL	Porphyrin	80 mg/g	1.5 h	LED 635 nm	37 J/cm^2^	MAL (80 mg/g)-PDT may be promising for the treatment of severe acne vulgaris.
Ma et al., 2015	Clinical	ALA	Porphyrin	5%	1 h	LED 633 nm	90–96 J/cm^2^	The therapy was effective for acne vulgaris in adolescents and the adverse effects were moderate and reversible.
Tao et al., 2015	Clinical	ALA	Porphyrin	3.6%	1.5 h	LED 633 nm	126 J/cm^2^	PDT was effective for the treatment of moderate to severe acne in Chinese patients and showed mild and transient adverse effects.
Tao et al., 2016	Clinical	ALA	Porphyrin	3.6%	1.5 h	LED 633 nm	126 J/cm^2^	ALA-PDT was efficient and safe for the treatment of severe acne vulgaris.
Yew et al., 2016	Clinical	ALA	Porphyrin	5%	3 h	Red light 630 nm	37 J/cm^2^	PDT was efficient and showed few adverse effect.
Ma et al., 2016	Ex vivo and in vitro	ALA	Porphyrin	5%	2 h	LED 633nm	96-108 J/cm^2^	PDT inhibited the innate immune response in *P. acnes*-infected keratinocytes via TLRs pathway
Ryu and Lee 2017	In vitro	Chlorin -e6	Chlorin	0.05, 0.1, 0.15, 0.2 μM	30 min	Halogen light	uninformed	Was observed an increase in collagen production by chlorin-mediated PDT, suggesting its potential use for scar amelioration and skin rejuvenation in acne treatment.
Kim et al., 2017	Randomized and comparative	MAL	Porphyrin	160 mg/g	30 min	Ablative 1550 nm fractional erbium glass laser	20 mJ/cm^2^	Daylight-PDT associated with MAL showed clinically good responses to inflammatory lesions in patients with moderate to severe acne.
Wang et al., 2017	In vitro and in vivo	Chlorin-e6	Chlorin	0.1, 0.5 e 1 μM	30 min	Halogen light	uninformed	Chlorin-e6-PDT suppressed *P. acnes-*induced inflammation through modulation of NFκB and MAPKs signaling pathways.
Wang et al., 2017	In vivo	ALA	Porphyrin	50%	2 h	Red light 630 nm (optical Intra-tissue fiber irradiation)	4.5 J/cm^2^	The intra-tissue irradiation presented few adverse effects than the conventional irradiated group, ALA. The treatment of acne in the ears of rabbits using intra-tissue irradiation showed better results on day 14, but not on days 30 and 45.
Qureshi and Lin 2017	Clinical	ALA	Porphyrin	20%	1–3 h	Red laser	50–100 J/cm^2^	The combination of non-ablative fractionated laser and PDT could be used in the treatment of acne, causing minimal side effects and requires fewer sessions than PDT alone, probably due to the increased ALA distribution caused by the pretreatment of the skin with the non-ablative fractionated photothermolysis.
Li et al., 2018	Case report	ALA	Porphyrin	3%	3 h	LED 633 nm	50 J/cm^2^	This method was effective after two years of treatment, and the presence of papules, without cysts and nodules, was reported.
De Annunzio et al., 2018	In vitro	Chlorin-e6	Chlorin	2.62, 5.25, 10.5, 21, 42 μM	10 min	LED 660 nm	3.25, 7.5 and 15 J/cm^2^	PDT was able to reduce the total microbial load in planktonic phase of *P. acnes*.

**Table 3:** Descriptions of studies using porphyrins and phthalocyanines in the treatment of psoriasis.

First Author and Year	Type of Study	PS	Chemical Family	Concentration (PS)	Pre-Irradiation Time	Light Source	Dose of Light	Results
Fransson e Ros, 2005	Clinical	ALA	Porphyrin	Freshly made cellulose gel (sodium carboxymethylcellulose in sterile water) containing 20% δ-ALA hydrochloride	Once weekly of two to five aplications.	Red light (maximum 630 nm) )	10–30 J/cm^2^	ALA treatment decreased the number of lesions.
Carrenho et al., 2015	in vivo	5,10-diphenyl-15,20-di(*N*-methylpyridinium-4-yl)porphyrin (Di-*cis*-Py+).	Porphyrin	Di-*cis*-Py+ (0.1 mg/ear)	20 min	Under white light from a compatible fiber optic probe (400–800 nm)	120 J/cm^2^	PDT reduced epidermis hyperproliferation, edema, proinflammatory cytokines, and cellular infiltration.
Jin et al., 2015	in vivo	Zinc phthalocyanine polymer conjugate (ZPB) with the polyethylene glycol (PEG) chain	Phthalocyanine	0.1 mg/mL and 1 mg/mL	6 days	UV-vis absorption wavelengths, 348 nm and 678 nm	uninformed	PDT showed good anti-psoriasis activity, based on the light excitation of the photosensitizer ZPB
Soler et al., 2016	in vitro	Silicon phthalocyanine (Pc) 4	Phthalocyanine	50, 100, 150, and 300 nM.	2 h	Red light (675 nm)	200 J/cm^2^	The mechanism of action is related to the generation of singlet oxygen and reactive oxygen species, leading to cell death through apoptosis mechanism in a dose response manner.
Liu et al., 2017	in vitro	α-(8-quinolinoxy) zinc (ZnPc-F7)	Phthalocyanine	0,10, 0,30 e 1,00 μg/mL	2 days	Laser 630 e 670 nm	14.15 J /cm^2^	The phthalocyanine derivative α- (8-quinolinoxy) zinc (ZnPc-F7), excited at 670 nm, reaches safely the deeper layers of the skin.

**Table 4:** The studies that used porphyrins in the treatment of HPV viral warts.

First Author and Year	Type of Study	PS	Chemical Family	Concentration (PS)	Pre-Irradiation Time	Light Source	Dose of Light	Results
Smucler; Jatsová, 2005	Clinical	ALA	Porphyrin	20%	3 h	Pulsed dye laser (PDL) 595 nm and LED 635 nm	20 and 50 J/cm^2^	The combination of PDT and PDL were able to cure infectious warts.
Wang et al., 2007	Clinical	ALA	Porphyrin	20%	4 h	Red light 590–700 nm	50 J/cm^2^	Noninvasiveness, minimal adverse effects, and efficient cosmetic results make PDT as a promising alternative treatment for recalcitrant viral warts in Asian population.
Fernandéz-Guarino; Harto; Jaén; 2011	Clinical	MAL	Porphyrin	160 mg/g	3 h	PDL 595 nm	9 J/cm^2^	PDL -PDT was effective and a tolerable treatment option for recalcitrant viral warts.
Li et al., 2014	Clinical	ALA	Porphyrin	5, 10, and 20%	4 h	Red light 633 nm	113 J/cm^2^	ALA (10%)-PDT was efficacious and safe for the treatment of recalcitrant facial verruca plana.
Qian et al., 2014	Clinical	ALA	Porphyrin	5, 10, and 20%	1.5 and 3 h	LED 630 nm	126 J/cm^2^	The efficacy and safety of PDT for the treatment of viral wart were dependent on the number of sessions and therapy doses.
Yang et al., 2016	Clinical	ALA	Porphyrin	10%	3 h	LED 630 nm	Uninformed	Three sessions: The optimal PDT scheme for the treatment of recalcitrant flat warts on the face of Chinese patients.

**Table 5:** Description of studies using porphyrins, phthalocyanines, and chlorins in the treatment of cutaneous leishmaniasis.

First Author and Year	Type of Study	PS	Chemical Family	Concentration (PS)	Pre-Irradiation Time	Light Source	Dose of Light	Results
Kosaka et al., 2007	In vitro and in vivo	ALA	Porphyrin	0.1 μm	4 h	Uninformed	50 J/cm^2^	ALA-PDT reduced the parasite load in mice.
Evangelou et al., 2011	The study conducted with a 69-year-old patient with a relapse of long-standing cutaneous leishmaniasis.	ALA	Porphyrin	Solution of 20% aminolevulinic acid in water	Three times at weekly intervals for three months.	Red light (light emitting diodes, 630 nm)	0.2 mL/cm^2^	Interaction of the PS with the light source reduced parasite load through generation of reactive oxygen species.
Mateus et al., 2014	In vitro *Leishmania* (Viannia) panamensis or *Leishmania (Leishmania) infantum* (*Leishmania chagasi*) parasites.	ALA	Porphyrin	0.0003–0.3 mM	48 h	597–752 nm	2.5 J/cm^2^	PSs is sub-localized into the host mitochondria and it was proposed that intracellular parasite elimination might be due to the death of the hosts cells.
Silva et al., 2015	In vitro in *L. amazonenses and Leishmania braziliensis*	PcZn_4_So_3_H -Zinc phthalocyanines	Phthalocyanines	1–10 μM	For 3 h and then exposed and incubate with Leishmania for 24 h	LED device at 660 nm	50 J/cm^2^	All PcZns exhibited high photoactivity at 10 μM.
Al-Qahtani et al., 2016	Assays with *Leishmania tropica* promastigotes and axenic amastigotes in vitro	Silicon(IV)-phthalocyanines	Phthalocyanines	1 μM	2 days	Red light (wavelength ∼650 nm)	2 J cm-^2^	Silicon (IV) –phthalocyanine derivatives presented antileishmanial effect and low citotoxicity to the host cells.
Pinto et al., 2016	In vitro study in *L. major* and *L. braziliensis* promastigotes.	chlorin-e6	Chlorin	400 μg/mL to 6.25 μg/mL	1 h	Biotable LED (Biopdi, 450 nm)	10 J/cm^2^	The authors alleged that PDT and chlorin-e6 internalization are disturbing the parasite mitochondrial activity; however, other assays are still missing such as O_2_ consumption and evaluation on parasites intracellular forms.
Ribeiro et al., 2016	Treatment of cutaneous leishmaniasis caused by *Leishmania* (*L. amazonensis*) in C57BL/6 mice	Liposomal chloroaluminium phthalocyanine (AlClPC)	Phthalocyanines	Oral Miltefosine200 mg/kg/day and PDT with topical AlClPC	20 days on alternate days.	Diode laser (BWF light) at 670 nm	80 mW	Combined miltefosine and PDT therapy using AlClPC as PS reduced the parasite load.
Viana et al., 2018	In vitro and in vivo	ALA	Porphyrin	0.1–10 µM	24–48 h	Red light	1–2 J/cm^2^	The PS amino-PC induced photo-inactivation of *L. amazonensis* in a dose-depedent manner.

**Table 6:** Description of the studies using porphyrin derivatives in skin rejuvenation.

First Author and Year	Type of Study	PS	Chemical Family	Concentration (PS)	Pre-Irradiation Time	LIGHT SOURCE	Dose of Light	Results
Clementoni et al., 2010	Clinical	ALA	Porphyrin	20%	1 h	Broadband Pulsed Light 560 nm and LED 630 nm and	19–22 J / cm^2^ and 75 J / cm^2^	Moderate improvement in fine lines, tactile roughness and firmness of the skin was observed in most patients.
Xi et al., 2011	Clinical	ALA	Porphyrin	5% and 10%	1 h	IPL 520–1200 nm	14–20 J/cm^2^ or 17–20 J/cm^2^	ALA-IPL has better efficacy in the skin rejuvenation of Chinese patients than IPL alone.
Jang et al., 2013	In vitro	ALA	Porphyrin	0.05–1 mM	30 min.	Incoherente light 633 nm	3–10 J /cm^2^	Intracellular EROs stimulated by PDT in dermal fibroblasts lead to the prolonged activation of ERK contributing to the effect of rejuvenation in PDT.
Ji et al., 2014	Clinical	ALA	Porphyrin	5, 10, and 20%	2 h	LED 630 nm	90 J/cm^2^	ALA-PDT was better than red light for skin rejuvenation.
Zhang et al., 2014	Clinical	ALA	Porphyrin	5%	2 h	Intense pulsed laser 550–570 nm and LED 630 nm	60 J/cm^2^	PDT combining two light sources was better for rejuvenation than the use of an individual light.
Friedmann et al., 2015	Clinical	ALA	Porphyrin	20%	1 h	Red light 635 nm, blue light 417 nm, intense pulsed light 560 nm, pulsed dye laser 595 nm	10, 37, (15–22), 6.5–8 J/cm^2^	No significant differences in skin rejuvenation and adverse effects were reported among the evaluated groups (blue light + PDL)/(blue light + IPL)/(blue light + PDL + IPL)/(blue light + red light + PDL + IPL).
Shin et al., 2015	Clinical	ALA	Porphyrin	0.5%	Applications at 5-min intervals for one hour	Intense pulsed light (400–720 nm) and long pulsed Nd: YAG laser	10.5 J/cm^2^ and 61.8–83.9 J/cm^2^	Liposomal spray-PDT reduced wrinkles without side effects, suggesting it as a promising alternative treatment for wrinkles in Asians.
Zhou et al., 2016	In vitro	ALA	Porphyrin	0.25, 0.50, 1.00 or 2.00 mmol/L	2 h or 6 h	laser 635 nm	25, 50 e 100 J/cm^2^	ALA-PDT causes oxidative damage and apoptosis in vitro in photoaged fibroblasts. This finding may be the basis for the rejuvenating effects of PDT on photoaged skin.
Kim et al., 2018	Clinical	MAL	Porphyrin	160 mg/g	3 h	LED 630 nm	37 J /cm^2^	PDT reduced mottled hyperpigmentation in photo aged patient skin, leading to skin whitening.

## Abbreviations

PDT	photodynamic therapy
PS	photosensitizers
ROS	reactive oxygen species
UV	ultraviolet radiation
PUVA	combination therapy using psoralen and UVA
HPV	human papillomavirus
CL	Cutaneous leishmaniasis
ALA	5-aminolevulinic acid
MAL	methyl aminolevulinate
PPIX	protoporphyrin IX
AlClPC	chloralaluminum phthalocyanine liposome
PDL	pulsed dye laser
IPL	intense pulsed light
